# Identification of a rare copy number polymorphic gain at 3q12.2 with candidate genes for familial endometriosis

**DOI:** 10.61622/rbgo/2024CR12

**Published:** 2024-03-15

**Authors:** Flávia Gaona Oliveira, Júlio Cesar Rosa-e-Silva, Alexandra Galvão Gomes, Juliana Dourado Grzesiuk, Thiago Vidotto, Jeremy Andrew Squire, Rodrigo Alexandre Panepucci, Juliana Meola, Lúcia Martelli

**Affiliations:** 1 Universidade de São Paulo Ribeirão Preto Medical School Department of Genetics Ribeirão Preto SP Brazil Department of Genetics, Ribeirão Preto Medical School, Universidade de São Paulo, Ribeirão Preto, SP, Brazil.; 2 Universidade de São Paulo Ribeirão Preto Medical School Department of Gynecology and Obstetrics Ribeirão Preto SP Brazil Department of Gynecology and Obstetrics, Ribeirão Preto Medical School, Universidade de São Paulo, Ribeirão Preto, SP, Brazil.; 3 Universidade de São Paulo Blood Center Center for Cell Therapy Ribeirão Preto SP Brazil Center for Cell Therapy, Blood Center, Universidade de São Paulo, Ribeirão Preto, SP, Brazil.

**Keywords:** Endometriosis, Endometrium, Heredity, Array-CGH, Genomic structural variation, DNA copy number variations, Polymorphism, genetic

## Abstract

Endometriosis is a complex disease that affects 10-15% of women of reproductive age. Familial studies show that relatives of affected patients have a higher risk of developing the disease, implicating a genetic role for this disorder. Little is known about the impact of germline genomic copy number variant (CNV) polymorphisms on the heredity of the disease. In this study, we describe a rare CNV identified in two sisters with familial endometriosis, which contain genes that may increase the susceptibility and progression of this disease. We investigated the presence of CNVs from the endometrium and blood of the sisters with endometriosis and normal endometrium of five women as controls without the disease using array-CGH through the Agilent 2x400K platform. We excluded common CNVs that were present in the database of genomic variation. We identified, in both sisters, a rare CNV gain affecting 113kb at band 3q12.2 involving two candidate genes: *ADGRG7* and *TFG*. The CNV gain was validated by qPCR. *ADGRG7* is located at 3q12.2 and encodes a G protein-coupled receptor influencing the NF-kappaβ pathway. *TFG* participates in chromosomal translocations associated with hematologic tumor and soft tissue sarcomas, and is also involved in the NF-kappa B pathway. The CNV gain in this family provides a new candidate genetic marker for future familial endometriosis studies. Additional longitudinal studies of affected families must confirm any associations between this rare CNV gain and genes involved in the NF-kappaβ pathway in predisposition to endometriosis.

## Introduction

Endometriosis is a disorder in which endometrial tissue grows outside the uterus, mainly on the pelvic peritoneum, but also on the ovaries and in the rectovaginal septum, and more rarely in the pericardium, pleura, and brain.^(1)^ Despite being an indolent lesion, endometriosis presents some characteristics of malignant diseases, such as uncontrolled proliferation, cell invasion, and neoangiogenesis.^(2,3)^ The main symptoms are pelvic pain, dysmenorrhea, and infertility, whereas some cases are asymptomatic. The prevalence of the disease has been hard to establish due to the invasive methods required to make a definitive diagnosis. Some studies show that endometriosis affects 10-15% of women in the reproductive age, and is diagnosed in 25-50% of infertile women and 30-80% of women with pelvic pain.^(4)^

Based on the analysis of twins and families, endometriosis is strongly associated with genetic factors.^(5-7)^ Studies of familial disease have shown that 4.8-5.8 and 4.9-8.1% of patients will have a sister or mother respectively affected by endometriosis.^(8,9)^ Furthermore, familial cases usually exhibit more severe disease and first-degree relatives have a six-fold risk of developing endometriosis.^(10)^ Studies with monozygotic twins showed that 87% of the sisters presented endometriosis, with 56% displaying moderate to severe disease.^(6)^

Genetic variation ranges in size from large microscopically visible chromosome anomalies to single nucleotide polymorphisms (SNPs). Copy Number Variation (CNVs) are structural alterations resulting in a variation in DNA copy numbers so that some regions may have more or fewer copies than the reference genome.^(11)^ The phenotypic effect of changes in the number of copies of a CNV is poorly understood.

In recent years, array technology has identified CNVs in ~12% of the human genome that may comprise several hundred kilobase pairs. CNVs contribute significantly to the inter-individual differences in humans, and CNV regions can range between 0.5 and 1.5 Mb among different genomes, typically involving duplication or deletion of affected regions.^(12)^ The contribution of the CNVs to genetic predisposition is not clear; however, studies show a correlation between the presence of CNVs affecting specific genes and a higher susceptibility to some diseases.^(11)^ In some physiological situations, CNVs are thought to inﬂuence the ability of normal cells to respond to changes in the microenvironment that could serve as an adaptive strategy for diverse cellular responses, such as hypoxia, metabolic deprivation, inflammatory responses, or cell survival and proliferation.^(13)^ Such effects of CNVs presumably come from changes in expression levels either because their altered DNA copy number directly effects on gene expression or through an indirect position effect on adjacent genes in the region.^(14)^ CNVs involving promoter regions of genes have been shown to influence gene expression and contribute to the development of complex disease traits.^(15)^

The impact of differential gene expression on downstream signaling pathways has been helpful in indicating disease processes that may be involved in endometriosis. There have been reported associations with regulation of disease susceptibility genes in several pathways, including inflammation, immune response, oxidative stress, hormone receptors and metabolism, matrix remodeling, cell adhesion, growth factors, cell cycle regulation, oncogenes and transcriptional regulation.^(16,17)^

Our working hypothesis for this study was that germline genomic CNVs found exclusively in familial cases and their first-degree relatives maybe involved in predisposition to endometriosis. We identified CNVs in two sisters with endometriosis and compared with the CNVs identified in five control women without disease. We then excluded common CNV polymorphisms detected by comparing each CNV to the database of genomic variation (DGV).^(12)^ We also compared the genomic alterations found in the endometrial samples with an accompanying matching blood sample to determine whether any detected CNVs were somatic alterations affecting endometrial tissues. Ultimately, we evaluated and correlated the role of the genes within each region of copy number alteration to the predisposition for developing endometriosis.

## Case description

The patients investigated are dizygotic twin sisters, with 35 years old, and were diagnosed with severe endometriosis by laparoscopy. We selected five women without infertility history or clinical symptoms of endometriosis and endometriotic lesions evidenced by laparoscopy during the tubal ligation procedure for the control group. The age of the control subjects varies between 27 and 38 years corresponding to the reproductive age.

The endometrium samples of the affected sisters and Control Group were biopsied using an endometrial suction catheter. We also collected 5 mL of blood of each participant of the study for comparison of DNA between endometrial and blood sample from the same participant so that we could distinguish between germline CNVs and somatic alterations in the endometrium leading to copy number change. After collection, the samples were stored in saline solution in -80ºC. The DNA from endometrial tissue and blood was extracted using the MasterPureTM Complete DNA and RNA Purification Kit – Epicentre (US) with 3mg of the tissue according to the manufacturer recommendations. Commercial control female DNA was used as reference control for array hybridization (Promega, Madison, WI). Array-CGH was performed with the Agilent 2x400K platform (Agilent, USA) which has 420.288 probes spaced by 5.3kb covering all the sample genome. The protocol followed the steps of DNA fragmentation, fluorescent labeling, probe hybridization, washing and signal scanning. The scanned fluorescence signs were analyzed through Nexus Copy Number 7.0 (BioDiscovery). We used the analysis algorithm Fast Adaptive States Segmentation Technique (FASST2) with linear significance of 1.0E-5 and the maximum between adjacent probes of 1000 kb to analyze the scanned fluorescence signs. Only alterations involving three or more consecutive overlapping probes were considered for analysis. Normal CNVs were considered in a log2 ratio between -0.62 and +0.42; values above +0.42 were evaluated as gain/amplification, above +1.14 as high gain, under -0.62 as losses (deletions) and those under -1.1 as homozygous loss.

We compared each alteration detected in our study with the stringent CNV map found in the DGV. The CNV map of the human genome catalogs benign CNVs among presumably healthy individuals of various ethnicities.^(12)^ The current map includes microscopic and submicroscopic variants from 50 bp to 3 Mb that are not associated with adverse phenotypes. The new CNV map from this study is presented as a standalone track in DGV (http://dgv.tcag.ca/dgv/docs/Stringent.Gain+Loss.hg19.2015-02-03.txt). This data was developed using selected recent high-resolution studies that were designed to maximize discovery and minimize false discoveries. Depending on the level of stringency of the map, we estimated that 4.8–9.5% of the genome contributes to CNV.^(12)^ This map has 11,762 alterations, and 10 are mapped to the Y chromosome. We excluded Y alterations considering 11,752 alterations related to the female samples.

The gain of the region chr3: 100,616,395 - 100,729,498 (GRCh38), identified by array-CGH was validated by qPCR. The assays were performed using *ADGR7* e *TFG* as target genes and the gene *TERT* as reference. The samples tested were: both sisters with the gain, the five women from control group and one woman with endometriosis without the gain.

The qPCR was performed in 7500 Real-Time PCR System (Applied Biosystems) and the reactions conditions were according recommendation of manufacturing. The qPCR was developed in final volume of 20µl, 2x TaqMan Genotyping Master Mix, Copy Number Assay, Copy Number Reference, water and 20ng of DNA. All reactions were performed in quadruplicate and analyzed on the Copy Caller Software. TaqMan^®^ Copy Number Assays ran simultaneously with a TaqMan^®^ Copy Number Reference Assay in a duplex, real-time PCR, using absolute quantitation settings. After amplification, the files were then imported into the CopyCaller™ Software for analysis of the copy number quantitation experiment.

We used Gene Card and PubMed databases to identify the function and correlation of the genes within the CNV identified with endometriosis.

All array data are available on the Gene Expression Omnibus (GEO http://ncbi.nlm.nih.gov/geo series record GSE85701).

This study was performed in the Molecular Cytogenetics Laboratory of the Genetics Department and the Gynecology and Obstetrics Department of the University of São Paulo and was approved by the ethics committee of the Clinical Hospital (HCRP 8559/2013). All the research subjects signed a written consent form which was approved by the ethics committee from Clinical Hospital of Ribeirão Preto.

## Results

**Figure 1:**
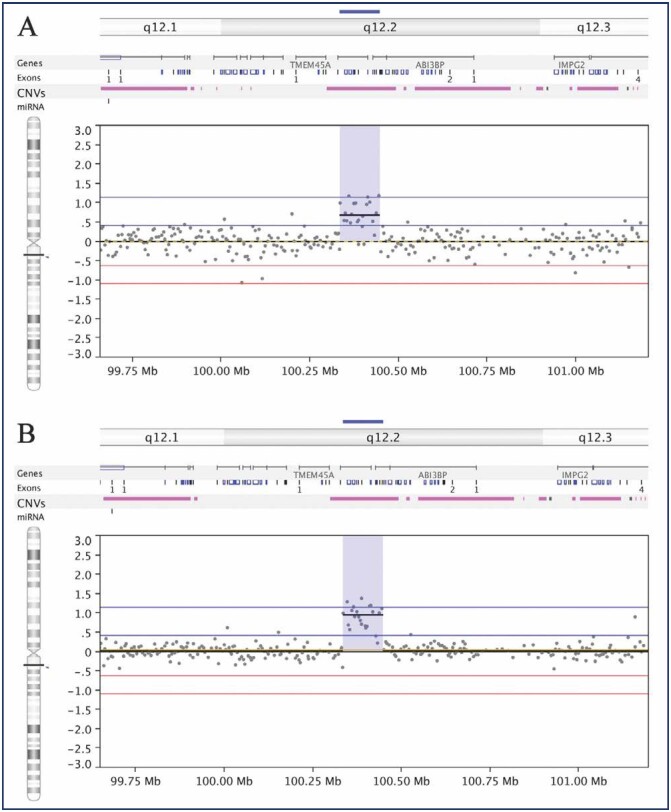
Genomic alterations detected by a-CGH in the sisters with endometriosis. Alterations detected in the chromosome 3. A – Gain in chromosome 3 from sister 1. B – Gain in chromosome 3 from sister 2

**Figure 2:**
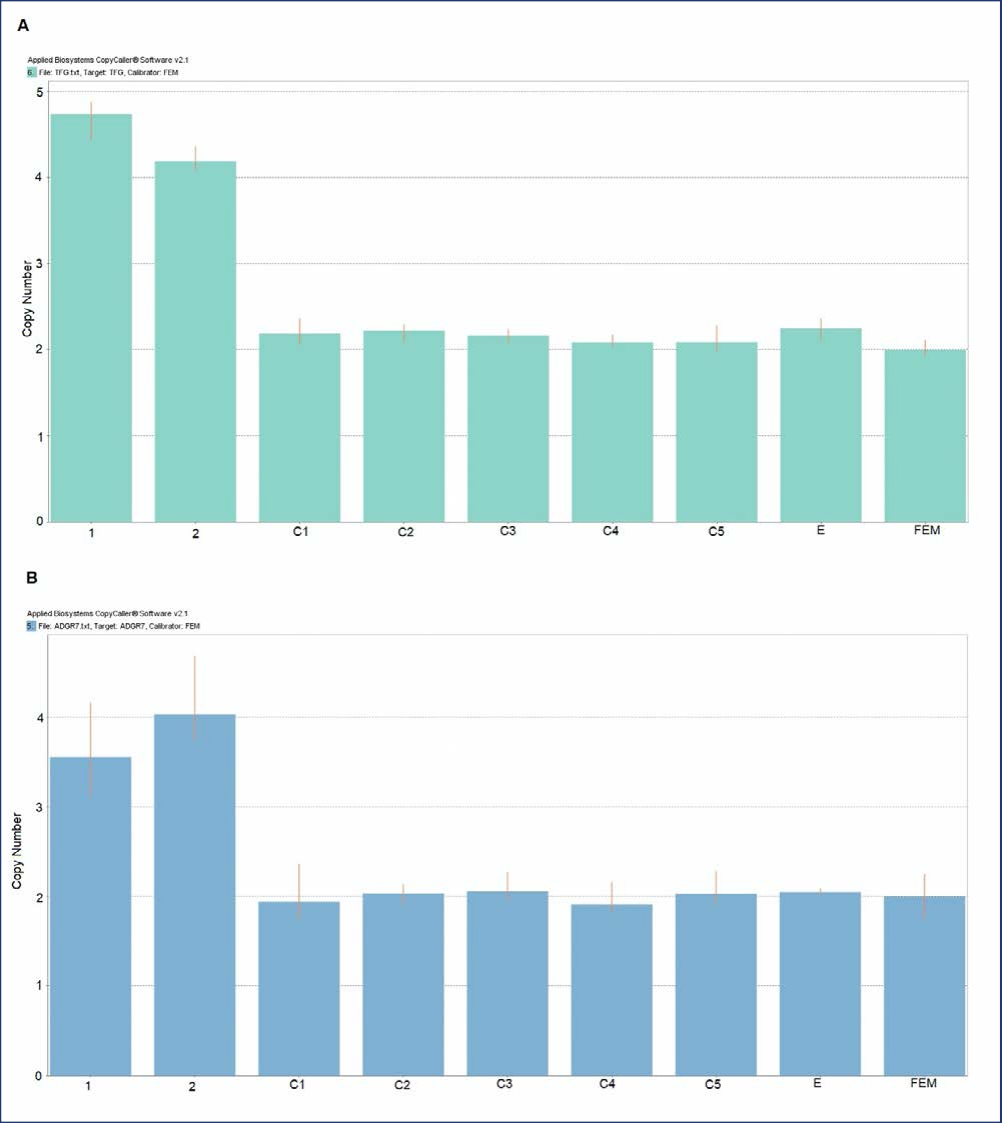
CNVs detected by qPCR validation. A – Gene TFG; B – Gene ADGR7. 1 and 2 = dizygotic twin sisters with endometriosis; C = control (without endometriosis); E = non-familial endometriosis; FEM = commercial control female DNA

The comparison between CNV alterations in endometrial tissue and blood DNA in the sisters, and the CNVs present in the control group allowed us to identify inherited CNVs that were potentially associated with the disease in the family being investigated. After exclusion of all CNVs shared with the Control Group or DGV database, one CNV region remained that was exclusive to the affected sisters. This CNV was present as a gain of 113,105bp at 3q12.2 in the interval 100,335,239-100,448,343 bp GRCh37, involving 22 probes. Two genes, *GPR128* and *TFG* (Figure 1), mapped to the location of CNV gain. In the figure 2, we show the results of validation assays confirming there was duplication of the genes within the CNV region.

To determine how common this CNV gain at 3q12.2 was in the general population we analyzed the overall incidence of this CNV gain in the DGV. We found that none of the 54,964 of the individuals from the DGV database exhibited this same CNV gain. There is another CNV closely linked to this region of gain at 3q12.2. This adjacent CNV is frequent in the DGV, occurring as a loss event at chr3:100,521,542-100,522,642, which is present in 0.29% (164/54,946) normal individuals, with 12% (20/164) being of Latin American ethnicity. Thus, the presence of this rare CNV gain at 3q12.2 in 2 sisters and its absence in the DGV suggests it is unlikely to be a common polymorphism, and may be considered an inherited CNV associated with predisposition to disease in this family.

## Discussion

Family studies show that relatives of the affected women have a higher risk to develop the disease, which suggest the influence of a genetic component in this process.^(18)^ Thus, currently, one of the main efforts for the researchers is to define the association between endometriosis and genetic polymorphisms. However, genes involved with the susceptibility to development and progression of endometriosis are still unknown.^(19)^ Moreover, no detailed study of the potential role of CNVs in familial endometriosis has been previously reported. This is the first genomic analysis of sisters with endometriosis to identify a rare CNV gain involving *ADGRG7* and *TGF* candidate genes. This CNV can be used as a new genetic marker for evaluation of familial endometriosis since the genes mapping to this region are involved in pathways associated with endometriosis. Since many of the CNVs are common polymorphisms that are not known to have disease association, it was then necessary to exclude these CNVs by comparison to the DGV.^(11)^

CNVs can influence the phenotype through loss of function, gain of function, gene dosage changes, and/or misregulation of genes within or near the CNV regions. When a polymorphic CNV is associated with DNA copy number losses, this change could lead to reduced expression of proteins so that haploinsufficiency of genes mapping to the CNV region may be a causative factor of phenotypic change. Similarly, the phenotypic consequences of duplications may arise because of increased expression of genes mapping within CNVs. In addition, the influence of CNVs on a given phenotype may depend not only on the relative position of genes within the CNV, but also on the proximity of closely linked genes to a breakpoint region of a *de novo* CNV.^(20)^

A recent analysis of CNVs in a small cohort of patients with sporadic endometriosis was unable to find an association with the disease etiology.^(21)^ However, the reported results were preliminary and a larger study group is required to examine the role of CNVs in this disease. Previous genome-wide association studies (GWAS) focusing on genomic alterations in endometrium of patients with endometriosis have shown various chromosomal alterations; however, only a few of these changes have been observed in more than one study.^(16,22,23)^ The inconsistency of the literature findings highlights the importance of getting robust and replicable results in order to identify the genetic influences on the disease traits.^(24)^

Many CNVs have been shown to be associated with diseases or increased susceptibility to conditions. The genetic mechanism leading to such association could be through gene dosage or a specific allelic combination in complex disorders.^(25)^ Henrichsen et al.^(25)^ showed that CNVs can not only affect gene expression of closely linked genes but may also have an influence on the transcriptome. They describe an overview of the impact of CNVs on gene expression patterns and suggest that these alterations play an even more important role with respect to normal phenotypic variation and risk to complex disease. Further studies are warranted for complete cataloging and fine mapping of CNVs, and elucidate all the mechanisms that could influence gene expression.

Furthermore, the chr3:100,335,239-100,448,343 region includes the genes *GPR128* and *TFG. GPR128* is the previous name for *ADGRG7*. There are several documented fusions of the *GPR128* and *TFG* genes that encode a hybrid oncoprotein.^(26)^ Ma et al.^(27)^ presented novel evidence that increasing frequency of fusion transcripts was associated with poor prognosis in cancer including TFG>GPR128. *TFG* gene itself also participates in several oncogenic rearrangements resulting in anaplastic lymphoma and myxoid chondrosarcoma, and may also play a role in the NF-kB pathway. NF-kB pathway-mediated gene transcription promotes inflammation, invasion, angiogenesis, and cell proliferation and inhibits apoptosis of endometriotic cells. NF-kB activation in macrophages and ectopic endometrial cells stimulates synthesis of proinflammatory cytokines, generating a positive feedback loop in the NF-kB pathway and promoting endometriotic lesion establishment, maintenance, and development.^(28)^ Because endometriosis is a multifactorial disease, inhibiting NF-kB seems to be a promising strategy for new therapies targeting different cell functions involved in endometriosis development, such as angiogenesis, cell adhesion, invasion, inflammation, proliferation, and apoptosis.^(29)^

The first cytogenomic study in patients with endometriosis was published by Gogusev et al.^(30)^ In this study, the authors analyzed the alteration in ectopic endometrium by CGH. Later, other genomic studies showed genomic alterations in eutopic, ectopic endometrium and blood and proposed a correlation between these alterations and the development of endometriosis.^(16,21,22,31-33)^

A major challenge in this field is that there is a growing number of CNVs of unknown significance that are suspected to be involved in disease susceptibility but for which additional population-level data are required.^(12)^ Since we examined 11,753 CNVs for association in this family we minimized the possibility of spuriously associating this CNV with disease by rigorously evaluating the function of gene candidates in the region for a potential role in endometriosis. Another limitation of this study is the small sample size of affected patients with this CNV. However, in view of the rarity of familial disease our findings suggest that additional longitudinal studies of affected families should be performed to confirm the occurrence of this rare CNV gain with predisposition to endometriosis.

The CNV described in this study was identified in both sisters and in both blood and endometrium samples, concluding that refers to an inherited alteration. Since the genes involved in the CNV region can be correlated to development of endometriosis, our study indicates, for the first time, that alterations found in familial samples may predispose the occurrence of the disease.

## Conclusion

The rare CNV gain involving *ADGRG7* and *TFG* genes in this family provides a new candidate genetic marker for future investigations involving familial endometriosis.
